# Investigation on GaN HEMTs Based Three-Phase STATCOM with Hybrid Control Scheme

**DOI:** 10.3390/mi12040464

**Published:** 2021-04-20

**Authors:** Chao-Tsung Ma, Zhen-Huang Gu

**Affiliations:** Department of Electrical Engineering, CEECS, National United University, Miaoli 36063, Taiwan; M0621002@smail.nuu.edu.tw

**Keywords:** wide bandgap (WBG), gallium nitride (GaN), high electron mobility transistor (HEMT), static synchronous compensator (STATCOM)

## Abstract

The modern trend of decarbonization has encouraged intensive research on renewable energy (RE)-based distributed power generation (DG) and smart grid, where advanced electronic power interfaces are necessary for connecting the generator with power grids and various electrical systems. On the other hand, modern technologies such as Industry 4.0 and electrical vehicles (EV) have higher requirements for power converters than that of conventional applications. Consequently, the enhancement of power interfaces will play an important role in the future power generation and distribution systems as well as various industrial applications. It has been discovered that wide-bandgap (WBG) switching devices such as gallium nitride (GaN) high electron mobility transistors (HEMTs) and silicon carbide (SiC) metal-oxide-semiconductor field-effect transistors (MOSFETs) offer considerable potential for outperforming conventional silicon (Si) switching devices in terms of breakdown voltage, high temperature capability, switching speed, and conduction losses. This paper investigates the performance of a 2kVA three-phase static synchronous compensator (STATCOM) based on a GaN HEMTs-based voltage-source inverter (VSI) and a neural network-based hybrid control scheme. The proportional-integral (PI) controllers along with a radial basis function neural network (RBFNN) controller for fast reactive power control are designed in synchronous reference frame (SRF). Both simulation and hardware implementation are conducted. Results confirm that the proposed RBFNN assisted hybrid control scheme yields excellent dynamic performance in terms of various reactive power tracking control of the GaN HEMTs-based three-phase STATCOM system.

## 1. Introduction

The static synchronous compensator (STATCOM) is a power electronic-based, shunt-type flexible AC transmission system (FACTS) device whose main functions include voltage regulation and reactive power flow control for power transmission, distribution, and industrial power supply systems. The STATCOM has become increasingly important because of the modern trend of renewable energy (RE) based distributed power generation (DG), where unpredictable fluctuations due to various weather conditions are unavoidable. Moreover, modern technologies such as Industry 4.0 and electric vehicles (EV) have various fast compensating requirements for their power utilization systems, including reactive power, unbalanced and harmonic currents, etc., which can be optimally dealt with using power electronic-based compensators [1,2,3,4].

In recent years, the investigation of STATCOM has always been very popular. Elserougi et al. [5] proposed a three-phase STATCOM based on a hybrid full-bridge (FB)/half-bridge (HB) 9-level boost modular converter with the aim of reducing the number of FB sub-modules. An interesting yes/no algorithm was proposed and its effectiveness was verified. In [6], a three-phase modular cascaded multilevel STATCOM consisting of conventional voltage source inverters (VSIs) was proposed with three independent DC links and an open-end winding transformer. The control was based on PI controller and the synchronous reference frame (SRF) theory. An isolated dual-converter-based three-phase distribution STATCOM (D-STATCOM) was proposed in [7]. The designed separate DC links mitigated undesired circulating currents. Optimal linear-quadratic controller was used for the required current control objectives.

As a shunt device, a STATCOM can be supported by energy storage systems (ESSs) and/or DG systems to perform functions such as optimal energy management and active power feeding. Liu et al. [8] investigated the abnormality in the control of a bridge converter-based STATCOM with battery ESS (BESS). A low pass filter (LPF) and a coordinated dual-loop active/reactive power control scheme were simulated using an electromechanical transient mathematical model. In [9], a cascaded 7-level H-bridge VSI-based grid-connected single-stage solar photovoltaic (PV)-STATCOM was presented with control strategy based on SRF theory and positive sequence detection algorithm. A particle swarm optimization (PSO)-based probabilistic voltage management scheme was used to investigate the allocation of PV-D-STATCOMs with and without ESSs and on-load tap changers for low- and mid-voltage distribution applications [10]. Wang et al. [11] used a STATCOM to improve the stability of a two-area power system consisting of a 19.8 MW onshore wind farm and a 100 MW offshore wind farm. A lead-lag power-oscillation damping controller was proposed. In [12], a self-excited induction generator (SEIG)-based wind farm and nonlinear three-phase and single-phase loads were interfaced with a STATCOM based on fixed capacitor bank and a state feedback-based control scheme, which allowed independent control of SEIG and capacitor bank voltages. A D-STATCOM with BESS, wind and solar PV generation systems was used in [13] with purpose of reducing voltage unbalance of a distribution system connected to a microgrid. The proposed sequence-component feedback controller significantly increased the stability of voltage and power outputs.

The core component of the STATCOM, D-STATCOM, and other power electronic-based FACTS devices is power semiconductor switching devices, such as metal-dioxide-semiconductor field-effect transistors (MOSFETs). In recent development trends, it is suggested that replacing conventional silicon (Si)-based power semiconductor switching devices with wide-bandgap (WBG) material-based ones in power converters can yield many merits, such as higher power rating, efficiency, switching frequency, and operating temperature. This is mainly because the WBG materials, including gallium nitride (GaN) and silicon carbide (SiC), possess superior properties including higher band gap, electric breakdown field, and saturated electron velocity. Moreover, GaN and SiC offer the highest electron mobility and thermal conductivity, respectively [14]. These properties allow the fastest and most efficient switching of the GaN high electron mobility transistors (HEMTs) and the SiC MOSFETs. Generally, GaN HEMTs are more suitable for low- to medium-voltage and low- to medium-power applications such as power flow controllers, power quality controllers, and EV charging applications, and SiC MOSFETs are more suitable for high-power and high-voltage applications such as various controllers for power transmission systems [15,16,17,18,19,20,21]. GaN HEMTs can be divided into three types, i.e., normally on depletion mode (D-mode), normally off enhancement mode (E-mode), and normally off cascode devices. The E-mode device offers lower conduction loss and has no body diode but has very strict driving voltage requirement (-10–7 V, threshold < 2 V); the cascode device offers less strict driving voltage requirement (±18 or ±20, threshold < 4V) but results in higher conduction loss, relatively lower operating temperature, and reverse recovery charge. The SiC MOSFET has similar structure to that of Si MOSFET yet almost an order thinner because of higher electrical breakdown field. The reduction in thickness results in smaller on-resistance (yet not as small as that of the GaN HEMT). Currently, the highest ratings of commercial devices are 1.7 kV/160 A for SiC MOSFETs, and 650 V/150 A (E-mode) and 900 V/34 A (cascode) for GaN HEMTs. [14,15,16,17,18,19,20,21]. 

However, successfully using the WBG switching devices requires avant-garde techniques for handling issues induced by the high slew rate of such devices. As a result, it is crucial to develop advanced driving circuits and optimize the printed circuit board (PCB) layout in order to obtain the best tradeoff between safety and losses [22,23]. For mid- to high-power applications, the requirements for driving WBG devices include the following: (1) high drive strength, (2) enough isolation between driving and power circuits (by using isolators or isolated drivers), (3) gate voltage oscillation damping (with high enough turn-on impedance and separated turn-on and turn-off paths), (4) gate voltage spike limiting (by using voltage clamps), fast turn-off (with small turn-off impedance and negative turn-off voltage) and (5) dead time optimization. For PCB layout, it is necessary to minimize parasitic inductance and capacitance. In other words, minimized trail lengths, device packages with small parasitic inductance, and minimized overlapping between paths are required [24,25,26].

Considering that there are currently no published articles discussing the use of GaN HEMTs for STATCOM design and applications, this paper aims to demonstrate a GaN HEMTs-based three-phase STATCOM and a hybrid control scheme for the first time. To explore the potential of improving dynamic control performance, the proposed GaN HEMTs-based STATCOM is based on a voltage-source inverter (VSI) topology and controlled with a dual-loop hybrid control scheme. Background knowledge of STATCOM and GaN HEMTs is briefly addressed in the first section. Section 2 establishes the proposed STATCOM system, including the architecture and relevant parameters. Section 3 presents the design of the proposed dual loop hybrid control scheme, including inner loop current controllers and outer loop DC link voltage and reactive power controllers. In this paper, a hybrid control structure using radial basis function neural network (RBFNN) controller alongside the PI controller is used in the reactive power control loop so that the dynamic performance of the GaN HEMTs-based STATCOM used as a fast reactive power tracking controller can be assessed. Simulation studies and experimental tests on a 2 kVA prototype are respectively presented in Section 4 and Section 5. A brief discussion and conclusion are given in Section 6.

## 2. System Description

**Figure 1:**
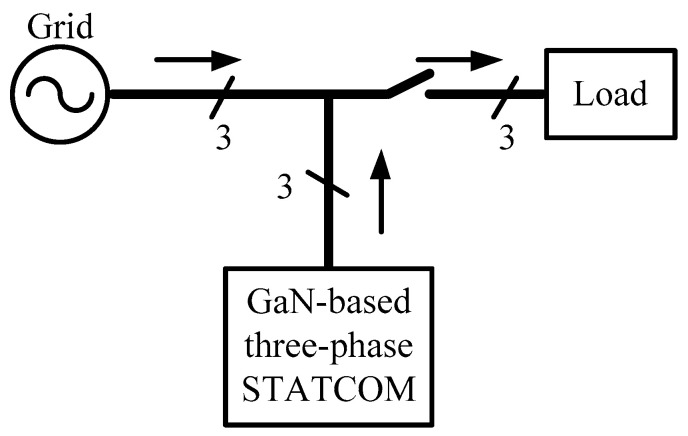
The test system architecture consisting of a grid, a load, and a GaN HEMTs-based three-phase STATCOM.

**Figure 2:**
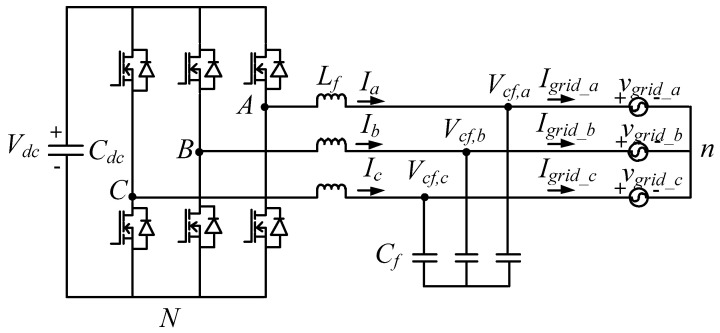
The circuit topology of the proposed GaN HEMTs-based STATCOM.

To focus on the control performance of the proposed GaN HEMTs-based STATCOM incorporated with different control schemes, the test system used in this study is simple, as shown in Figure 1. It consists of a healthy and balanced three-phase grid, a load, and a three-phase GaN HEMTs-based STATCOM. The three-phase STATCOM adopts a voltage-source inverter (VSI) architecture because of the simplicity, as shown in Figure 2, where $V_{dc}$ represents DC link voltage, $C_{dc}$ represents DC link capacitor, *A*, *B*, and *C* are the switching points of phase legs *A*, *B*, and *C*, respectively, *N* is the reference point of the VSI voltages, $L_{f}$ represents the filter inductor, $I_{a}$, $I_{b}$, and $I_{c}$ represent inductor currents, $C_{f}$ represents the filter capacitor, $V_{cf,a}$, $V_{cf,b}$ and $V_{cf,c}$ represents filter capacitor voltages, $I_{grid\_a}$, $I_{grid\_b}$ and $I_{grid\_c}$ represent grid currents, $V_{grid\_a}$, $V_{grid\_b}$, and $V_{grid\_c}$ represent grid voltages, and *n* represents grid grounding point. Relevant parameters are listed in Table 1. In this paper, the main control function of the GaN HEMTs-based three-phase STATCOM is to provide fast and precise reactive power regulation.

## 3. Controller Design

**Figure 3:**
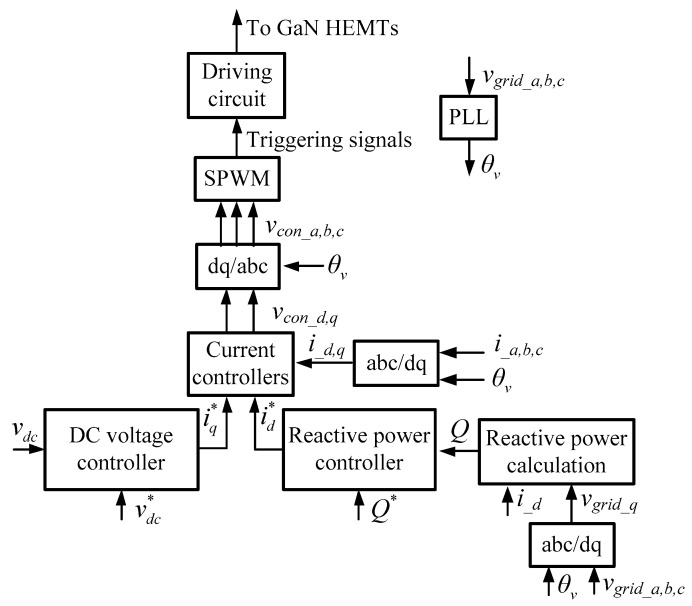
The proposed control architecture for GaN HEMTs-based three-phase STATCOM.

In theory, the reactive power output of a STATCOM can be controlled with its voltage phase shifts or various reactive current control schemes. Direct reactive current control is normally chosen because of the simplicity and easy to implement. To achieve a fast dynamic control feature, the control strategy of the proposed STATCOM is based on the dq-axis decoupling in SRF and a dual-loop architecture, as shown in Figure 3, where the inner loop consists of dq-axis type-II current controllers, and the outer loop consists of two PI controllers for both the reactive power (d-axis) and DC link voltage (q-axis). For comparison purposes, an RBFNN controller and a PI controller are designed for the reactive power control loop in which sinusoidal pulse width modulation (SPWM) is used to switch the GaN HEMTs.

### 3.1. Design of Inductor Current Controllers

For the design of inductor current controllers, the mathematical models of the inductor currents are obtained based on Figure 2 and Kirchhoff’s voltage law:

(1) $$L_{f}\frac{dI_{a}}{dt}=v_{AN}-v_{grid\_a}-v_{nN};$$

(2) $$L_{f}\frac{dI_{b}}{dt}=v_{BN}-v_{grid\_b}-v_{nN};$$

(3) $$L_{f}\frac{dI_{c}}{dt}=v_{CN}-v_{grid\_c}-v_{nN},$$

where $v_{AN}$, $v_{BN}$, and $v_{CN}$ represent switching point voltages, and **v_nN_** represents the voltage difference between grid ground and VSI neutral point. Assuming that the three phases of the VSI are balanced, we get the following:

(4) $$I_{a}+I_{b}+I_{c}=0.$$

As a result, *v_nN_* can be expressed as follows:

(5) $$v_{nN}=\frac{\left(v_{AN}+v_{BN}+v_{CN})-(v_{grid\_a}+v_{grid\_b}+v_{grid\_c}\right)}{3},$$

which allows (1)–(3) to be expressed as the following:

(6) $$\left[\begin{array}{l} L_{f}\frac{dI_{a}}{dt}\\ L_{f}\frac{dI_{b}}{dt}\\ L_{f}\frac{dI_{c}}{dt} \end{array}\right]=\frac{2}{3}\left[\begin{array}{l} 1\;\frac{-1}{2}\;\frac{-1}{2}\\ \frac{-1}{2}\;1\;\frac{-1}{2}\\ \frac{-1}{2}\;\frac{-1}{2}\;1 \end{array}\right](\left[\begin{array}{l} v_{AN}\\ v_{BN}\\ v_{CN} \end{array}\right]-\left[\begin{array}{l} v_{grid\_a}\\ v_{grid\_b}\\ v_{grid\_c} \end{array}\right]).$$

Since pulse width modulation (PWM) technique is used in the control, the three-phase modulation signals $v_{cona}$, $v_{conb}$ and $v_{conc}$ are compared with carrier signal $v_{tri}$ respectively to trigger the switches of the switching legs. As a result, $v_{AN}$, $v_{BN}$, and $v_{CN}$ can be expressed as follows:

(7) $$v_{AN}=(\frac{1}{2}+\frac{v_{cona}}{2v_{tri}})V_{dc};$$

(8) $$v_{BN}=(\frac{1}{2}+\frac{v_{conb}}{2v_{tri}})V_{dc};$$

(9) $$v_{CN}=(\frac{1}{2}+\frac{v_{conc}}{2v_{tri}})V_{dc}.$$

Substituting (7)–(9) into (6) and letting $\frac{V_{dc}}{2V_{tri}}=K_{pwm}$ yield the following:

(10) $$\left[\begin{array}{l} L_{f}\frac{dI_{a}}{dt}\\ L_{f}\frac{dI_{b}}{dt}\\ L_{f}\frac{dI_{c}}{dt} \end{array}\right]=\frac{2}{3}\left[\begin{array}{l} 1\;\frac{-1}{2}\;\frac{-1}{2}\\ \frac{-1}{2}\;1\;\frac{-1}{2}\\ \frac{-1}{2}\;\frac{-1}{2}\;1 \end{array}\right](K_{pwm}\left[\begin{array}{l} v_{cona}\\ v_{conb}\\ v_{conc} \end{array}\right]-\left[\begin{array}{l} v_{grid\_a}\\ v_{grid\_b}\\ v_{grid\_c} \end{array}\right]).$$

**Figure 4:**
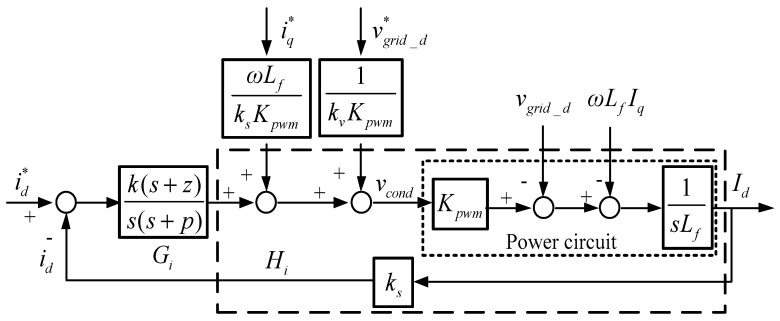
Control loop of d-axis current controller.

**Figure 5:**
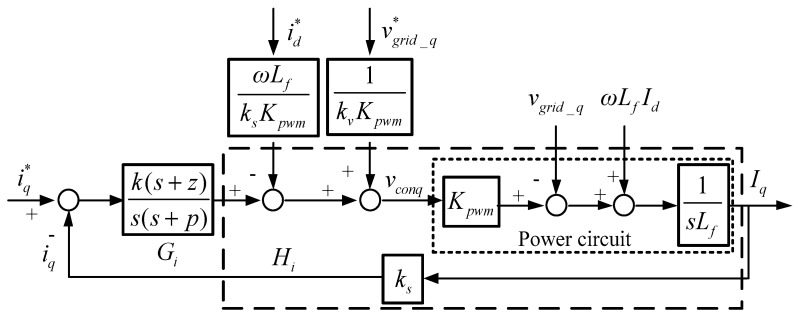
Control loop of q-axis current controller.

Next, (10) can be converted into dq0-axis equivalents using synchronous reference frame (SRF) theory:

(11) $$\begin{array}{c} \left[\begin{array}{c} L_{f}\frac{dI_{d}}{dt}\\ \begin{array}{l} L_{f}\frac{dI_{q}}{dt}\\ L_{f}\frac{dI_{0}}{dt} \end{array} \end{array}\right]=K_{pwm}\left[\begin{array}{ccc} 1 & 0 & 0\\ 0 & 1 & 0\\ 0 & 0 & 1 \end{array}\right]\left[\begin{array}{c} v_{cond}\\ v_{conq}\\ v_{con0} \end{array}\right]\\ -\left[\begin{array}{ccc} 1 & 0 & 0\\ 0 & 1 & 0\\ 0 & 0 & 1 \end{array}\right]\left[\begin{array}{c} v_{grid\_d}\\ \begin{array}{l} v_{grid\_q}\\ v_{grid\_0} \end{array} \end{array}\right]-\left[\begin{array}{ccc} 0 & \omega L_{f} & 0\\ -\omega L_{f} & 0 & 0\\ 0 & 0 & 0 \end{array}\right]\left[\begin{array}{l} I_{d}\\ I_{q}\\ I_{0} \end{array}\right]\; \end{array}$$

where *v_cond_*, *v_conq_*, and *v*_*con*0_ represent dq0-axis modulation signals, *v_grid_d_*, *v_grid_q_*, and *v*_*grid*_0_ represent grid dq0-axis voltages, and *I_d_*, *I_q_*, and *I*_0_ represents inductor dq0-axis currents. Then, block diagrams of dq-axis inductor current controllers can be graphed according to (11), as shown in Figure 4 and Figure 5, where type II controllers are used, i_d_ and i_q_ represent sensed dq-axis currents, $i_{d}^{*}$ and $i_{q}^{*}$ represent dq-axis current commands, and $v_{grid\_d}^{*}$ and $v_{grid\_q}^{*}$ represent qd-axis grid voltage commands. Then, we can obtain current loop transfer function, controller transfer function, and loop gain as follows:

(12) $$H_{i}(s)=\frac{k_{s}K_{pwm}\;}{sL_{f}};$$

(13) $$G_{i}(s)=\;\frac{k(s+z)}{s(s+p)};$$

(14) $$L_{i}(s)=G_{i}(s)H_{i}(s)=\;\frac{k(s+z)}{s(s+p)}\;\;\frac{k_{s}K_{pwm}\;}{sL_{f}}.$$

In practical applications, the crossover frequency of a type II controller is designed below 1/4 of the switching frequency. In this paper, the crossover frequency is designed at 1/10 of the switching frequency, the zero is designed at 1/5 of the crossover frequency, and pole is designed at 36.15 kHz:

(15) $$\omega_{i}=100k\times 2\pi /10=\;62832rad/s;$$

(16) $$z=\omega_{i}/5=1256.6rad/s;$$

(17) p=2π×36.15k=227140rad/s.

Next, current loop gain and controller gain at crossover frequency can be obtained:

(18) $$Gain_{Hi}=\frac{k_{s}K_{pwm}\;}{sL_{f}}=0-j0.0159\Rightarrow \left|Gain_{Hi}\right|=0.0159;$$

(19) $$Gain_{Gi}=\frac{\left(s+z\right)}{s(s+p)}=3.8634\times 10^{-6}-j1.9492\times 10^{-6}\;\Rightarrow \left|Gain_{Gi}\right|=4.3273\times 10^{-6}.$$

The required gain compensation at crossover frequency can then be calculated:

(20) $$k=\frac{1}{\left|Gain_{Hi}\right|\times \left|Gain_{Gi}\right|}=1.452\times 10^{7}.$$

As a result, controller transfer function is expressed as follows:

(21) $$G_{i}(s)=\;\frac{1.452\times 10^{7}(s+1256.6)}{s(s+227140)}.$$

**Figure 6:**
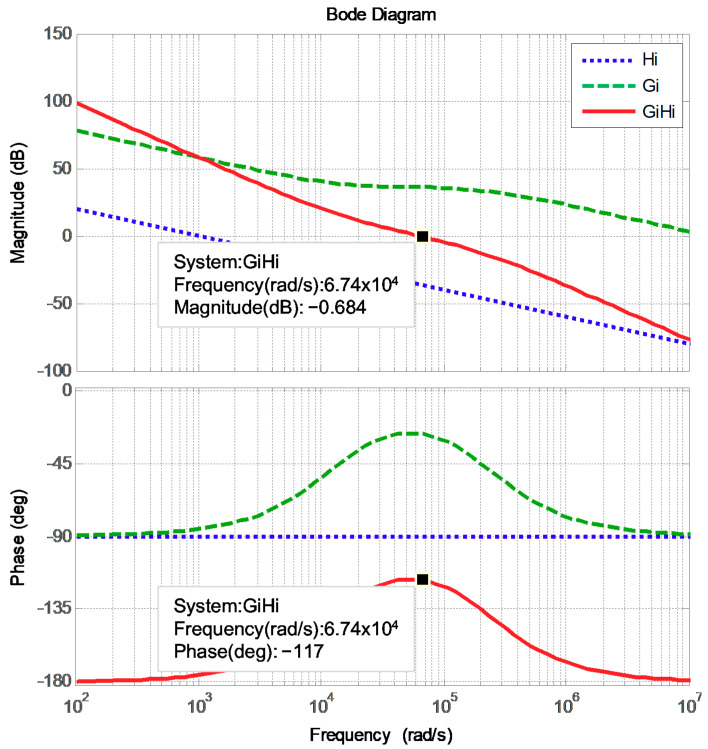
Bode plot of the two inductor current control loops.

The designed proportional and integral gains are 63.9257 and 8.033188, respectively. Figure 6 displays the Bode plot of the inductor current control loops, where the phase margin is 63 degrees.

### 3.2. DC Link Voltage Controller

**Figure 7:**
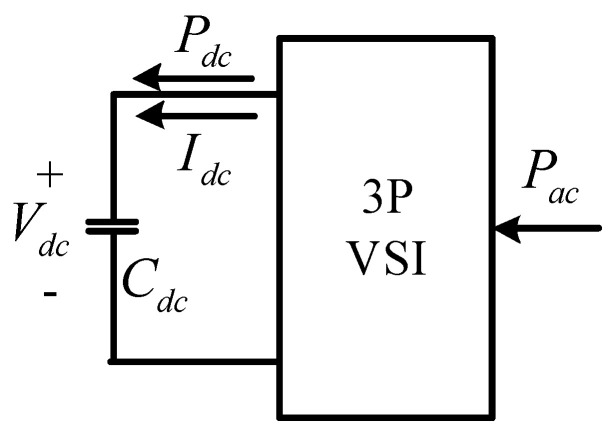
Equivalent circuit of VSI voltage loop.

The DC link voltage control loop balances the active power between AC and DC sides of the VSI. By neglecting steady-state operating point, we can obtain an equivalent circuit of the voltage loop as illustrated in Figure 7.

According to Figure 7, the instantaneous AC power at AC side in SRF can be defined as follows:

(22) $$P_{ac}=1.5v_{grid\_q}I_{q}.$$

Mapping AC side signals onto the DC side and assuming that the inverter is lossless, we obtain the following:

(23) $$P_{ac}=P_{dc};$$

(24) $$1.5v_{grid\_q}I_{q}=V_{dc}I_{dc}.$$

As a result, the relationship between DC side current and AC side current is as follows:

(25) $$I_{dc}=\frac{1.5v_{grid\_q}}{V_{dc}}I_{q}\;\;\;\;=k_{dc}I_{q\;\;};$$

(26) $$C_{dc}\frac{dV_{dc}}{dt}=I_{dc}\Rightarrow \;\;\;V_{dc}=\;\;\;I_{dc}\;\;\frac{1}{s\;C_{dc}}\;.$$

**Figure 8:**
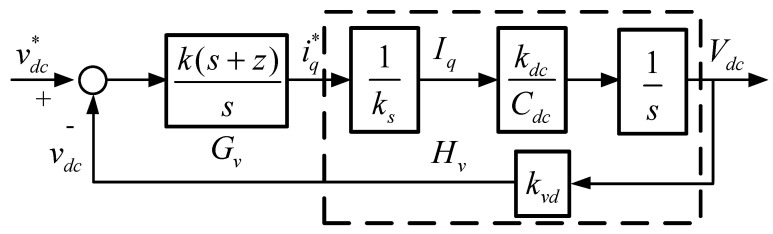
Control loop of DC link voltage controller.

According to (25) and (26), we can obtain the transfer function of DC side voltage:

(27) $$\;\frac{\;V_{dc}}{I_{q}}=\frac{k_{dc}}{s\;C_{dc}},$$

where *k_dc_* represents the conversion factor from AC side to DC side. This relationship yields the block diagram of DC link voltage control loop, as shown in Figure 8, where PI controller is used, and only q-axis current is controlled. Then, we can obtain DC link voltage loop transfer function, controller transfer function, and loop gain as follows:

(28) $$H_{v}(s)\;=\frac{k_{vd}k_{dc}}{k_{s}C_{dc}s};$$

(29) $$G_{v}(s)=\;\frac{k(s+z)}{s};$$

(30) $$L_{v}(s)=G_{v}(s)H_{v}(s)=\;\frac{k(s+z)}{s}\;\frac{k_{vd}k_{dc}}{k_{s}C_{dc}s}.$$

The main purpose of the PI controller is to reduce possible interference affecting the DC link voltage when the STATCOM regulates reactive power. Usually, the crossover frequency of the outer loop is designed below 1/3 of that of the inner loop. The crossover frequency is set at 1/50 of that of the current loop, and the zero is designed at 1/5 of DC loop crossover frequency:

(31) $$\omega_{v}=\omega_{i}/50=1256.6rad/s;$$

(32) $$z=\omega_{v}/5=251.328rad/s.$$

Next, DC link voltage loop gain and controller gain at crossover frequency can be obtained:

(33) $$Gain_{Hv}=\frac{k_{vd}k_{dc}}{k_{s}C_{dc}s}=0-j0.0946\Rightarrow \left|Gain_{Hv}\right|=0.0946;$$

(34) $$Gain_{Gv}=\frac{k(s+z)}{s}=1-j0.02\Rightarrow \left|Gain_{Gv}\right|=1.0198.$$

The required gain compensation at crossover frequency can then be calculated:

(35) $$k=\frac{1}{\left|Gain_{Hv}\right|\times \left|Gain_{Gv}\right|}=10.3673;$$

As a result, controller transfer function is expressed as follows:

(36) $$G_{v}(s)=\;\frac{10.3673(s+251.328)}{s}.$$

**Figure 9:**
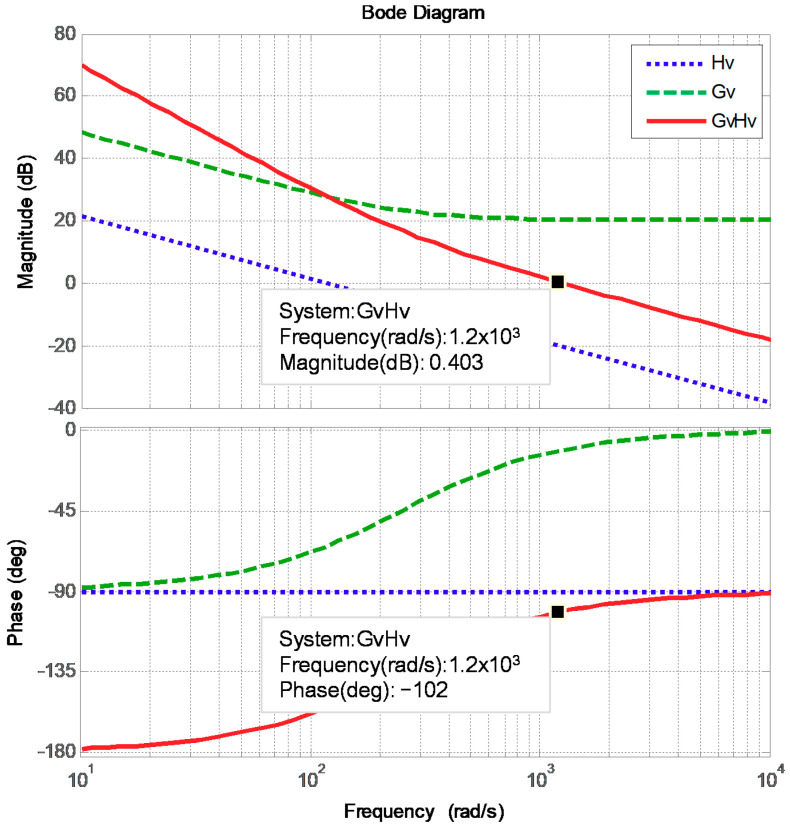
Bode plot of the DC link voltage control loop.

The designed proportional and integral gains are 10.3673 and 0.02591825, respectively. Figure 9 displays the Bode plot of the inductor current control loops, where the phase margin is 78 degrees.

### 3.3. Reactive Power Controller

The reactive power control loop regulates the reactive power output of the VSI. Instantaneous reactive power can be defined as follows:

(37) $$Q=-1.5v_{grid\_q}I_{d};$$

(38) $$\frac{Q}{I_{d}}=-1.5v_{grid\_q}.$$

**Figure 10:**
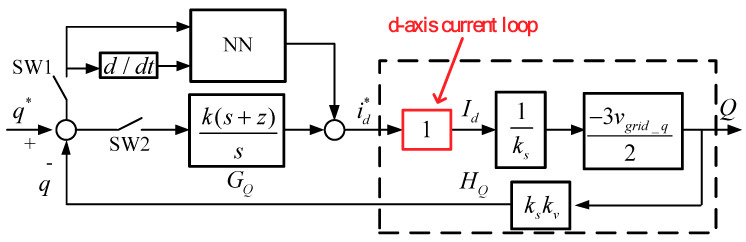
Control loop of reactive power controller.

As a result, the block diagram of reactive power control loop can be graphed as shown in Figure 10, where PI controller is used, only d-axis current is controlled, and *q* and q^*^ represent sensed reactive power and reactive power command, respectively. In this paper, an RBFNN controller is designed and added in the reactive power control loop. It is designed to improve the dynamic performance of the PI controller in fast tracking control of reactive power. By switching SW1 and SW2, the NN and PI controllers can be activated simultaneously or separately. It is assumed that the bandwidth of current control loop is much wider than that of the active/reactive power control loop. The addition of the RBFNN controller can help the linear PI controller deal with transients, but it naturally increases complexity and cost. We can obtain reactive power loop transfer function, controller transfer function, and loop gain as follows:

(39) $$H_{Q}(s)\;=\frac{-3k_{v}v_{grid\_q}}{2};$$

(40) $$G_{Q}(s)=\;\frac{k(s+z)}{s};$$

(41) $$L_{Q}(s)=G_{Q}(s)H_{Q}(s)=\;\frac{k(s+z)}{s}\;\frac{-3k_{v}v_{grid\_q}}{2}.$$

For the PI parameters, the crossover frequency is set at 1/150 of that of the current control loop, and the zero is designed at 10 times the crossover frequency:

(42) $$\omega_{q}=\omega_{i}/150=418.88rad/s;$$

(43) $$z=\omega_{q}*10=4188.8rad/s.$$

Next, reactive power loop gain and controller gain at crossover frequency can be obtained:

(44) $$Gain_{HQ}=\frac{-3k_{v}v_{grid}}{2}=0.8352-j0\Rightarrow \left|Gain_{Hdc}\right|=0.8352;$$

(45) $$Gain_{GQ}=\frac{k(s+z)}{s}=1-j10\Rightarrow \left|Gain_{GQ}\right|=10.0499.$$

The required gain compensation at crossover frequency can then be calculated:

(46) $$k=\frac{1}{\left|Gain_{HQ}\right|\times \left|Gain_{GQ}\right|}=0.1191;$$

As a result, controller transfer function is expressed as follows:

(47) $$G_{Q}(s)=\frac{0.1191(s+4188.8)}{s}.$$

**Figure 11:**
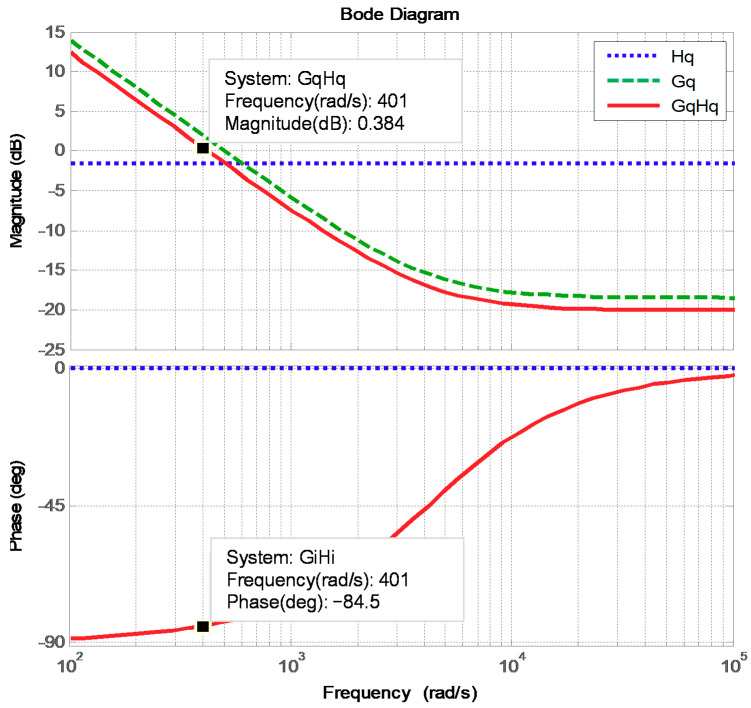
Bode plot of the reactive power control loop.

The designed proportional and integral gains are 0.1191 and 0.0049888996, respectively. Figure 11 displays the Bode plot of the inductor current control loops, where the phase margin is 95.5 degrees.

**Figure 12:**
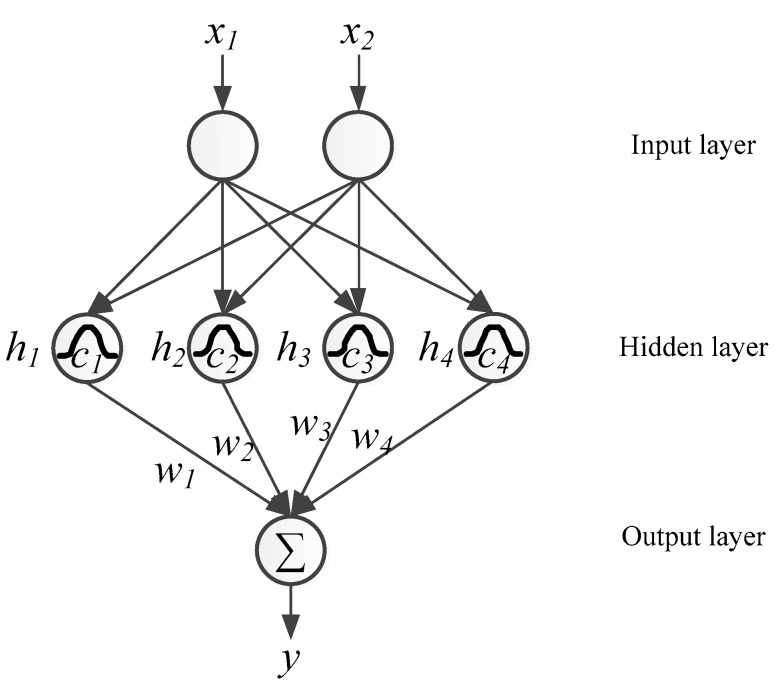
Proposed RBFNN configuration.

The RBFNN controller added in the reactive power controller loop is as shown in Figure 12. The RBFNN is a feedforward NN using Gaussian activation functions, and the weights of the RBFNN are determined adaptively. The RBFNN offers advantages such as structural and computational simplicity, high noise immunity, avoidance of local minima, and fast online learning. The RBFNN is widely used in applications such as parameter identification and pattern recognition [27,28,29]. In this paper, 2, 4, and 1 node are designed for the input, hidden, and output layers, respectively, determined with try and error within the computational limitation of the adopted digital signal processor (DSP) TMS320F28335 by Texas Instruments. The adopted RBFNN controller is trained with online learning.

## 4. Simulation Study

**Figure 13:**
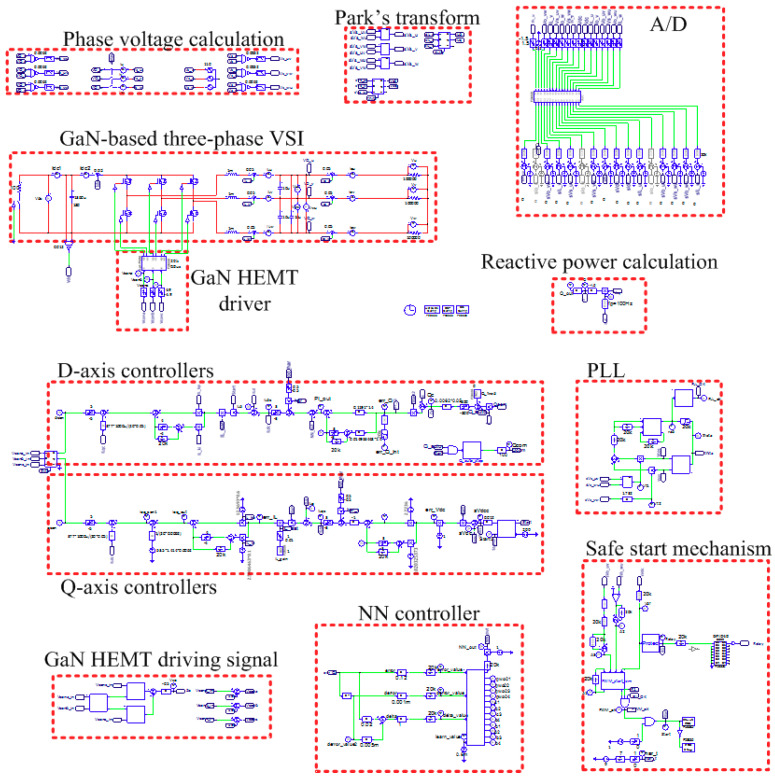
Simulation model in software environment.

**Figure 14:**
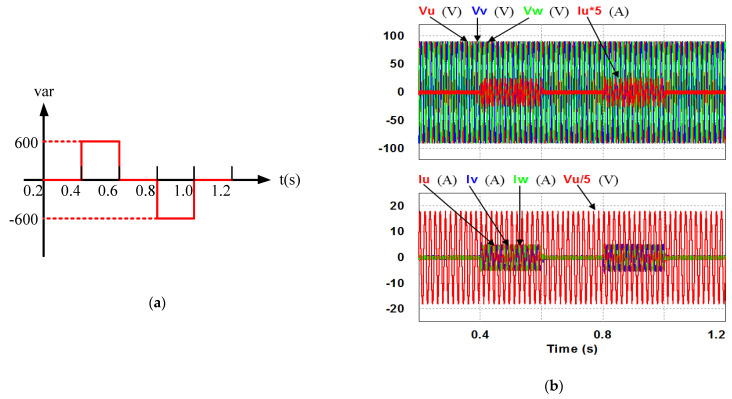
(**a**) Simulation scenario: sequence diagram of reactive power command; (**b**) case 1 simulation result: three-phase voltages and currents.

The simulation studies in this work were conducted using simulation software. The simulation environment is as shown in Figure 13. The control objective of the designed 2 kVA GaN HEMTs-based three-phase STATCOM is fast response and precise reactive power output based on a given reactive power command, as shown in Figure 14a. The command consists of three states: 0, 600, and −600 VAR, and each interval is 0.2 s long. For comparison of the designed reactive power controllers, two controller settings are arranged, as listed in Table 2. In the following results, the values of the control signals (command and feedback waveforms) are the actual values multiplied by their respective sensing factors and have been converted into voltage signals, as listed in Table 3. That is, the 600 VAR reactive power corresponds to 0.186V reactive power control signal, and the 3.15 A reactive current corresponds to 0.1575V reactive current control signal.

### 4.1. Results of Simulation Case 1: PI Controller

**Figure 15:**
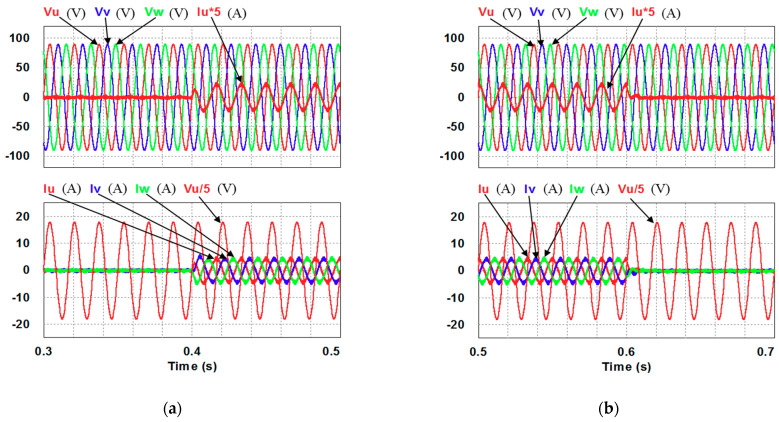
Detailed views of Figure 14b: (**a**) from 0.3 to 0.5 s; (**b**) from 0.5 to 0.7 s.

**Figure 16:**
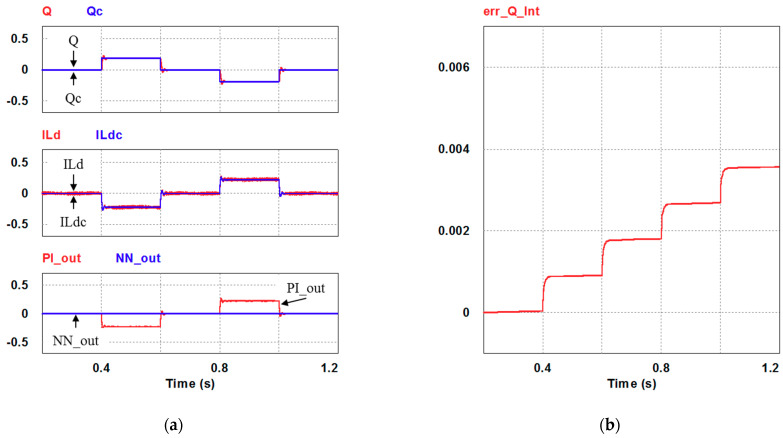
Case 1 simulation result: (**a**) reactive power feedback and its command, inductor current power and its command, and reactive power controller outputs (all control signals are in V); (**b**) the accumulated reactive power control error.

**Figure 17:**
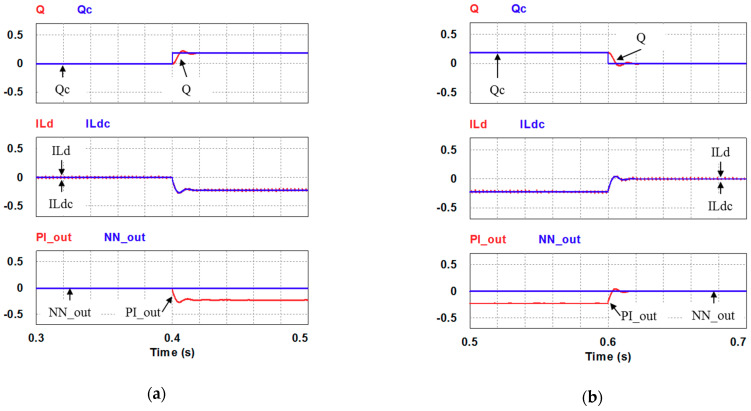
Detailed views of Figure 16a: (**a**) from 0.3 to 0.5 s; (**b**) from 0.5 to 0.7 s.

In this case, only the PI controller is activated in the reactive power control loop. Simulation results are presented in Figure 14b, Figure 15b and Figure 16b. Figure 14b and Figure 15 show that the phases and amplitudes of three-phase currents are well controlled. In Figure 16a and Figure 17, it can be observed that the rise and fall times for reactive power are 3.469 ms and 3.558 ms, respectively, and the overshoot and undershoot percentages are both 20%. In Figure 16b, it can be observed that the accumulated reactive power control error is 3.57m at the end of the simulation.

### 4.2. Results of Simulation Case 2: PI Controller + RBFNN Controller

**Figure 18:**
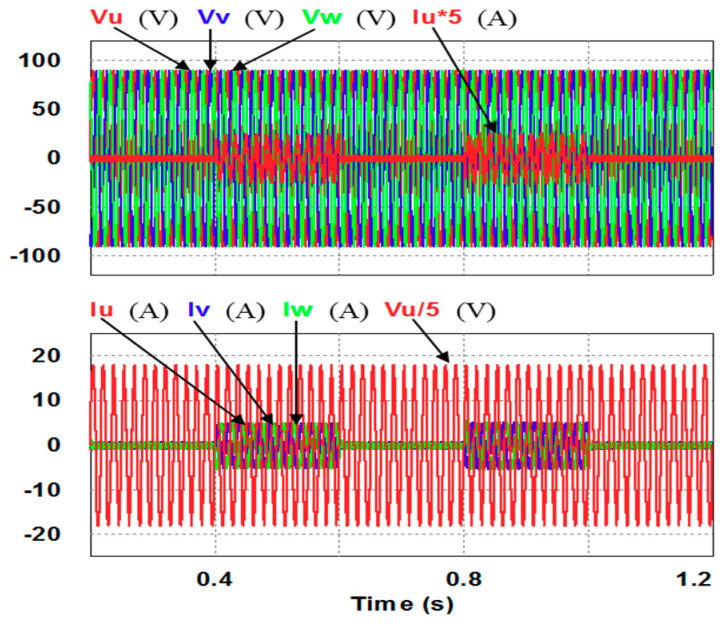
Case 2 simulation result: three-phase voltages and currents.

**Figure 19:**
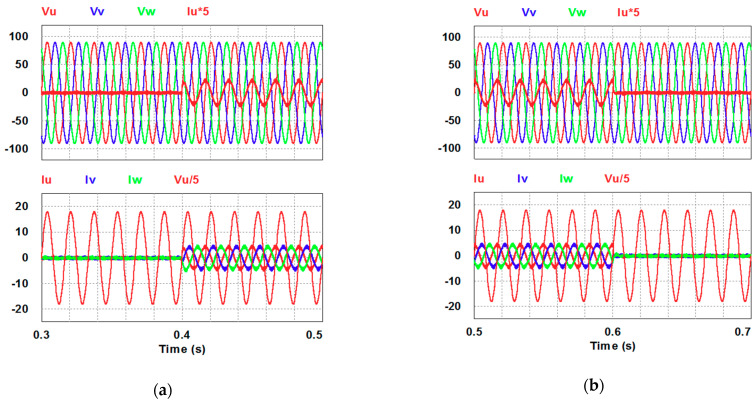
Detailed views of Figure 18: (**a**) from 0.3 to 0.5 s; (**b**) from 0.5 to 0.7 s.

**Figure 20:**
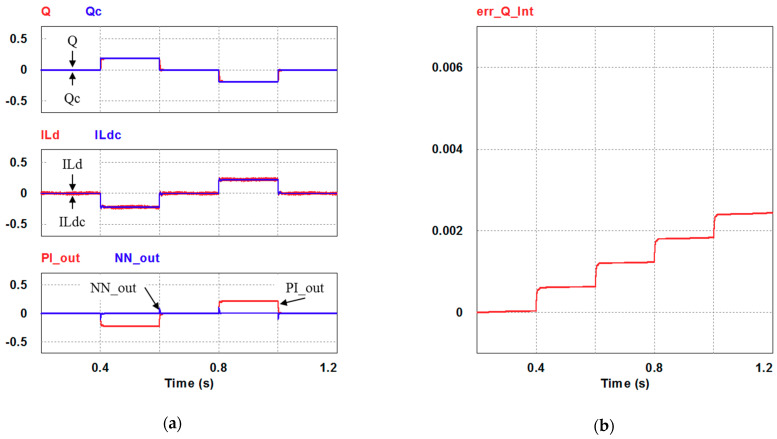
Case 2 simulation result: (**a**) reactive power feedback and its command, inductor current power and its command, and reactive power controller outputs (all control signals are in V); (**b**) accumulated reactive power control error.

**Figure 21:**
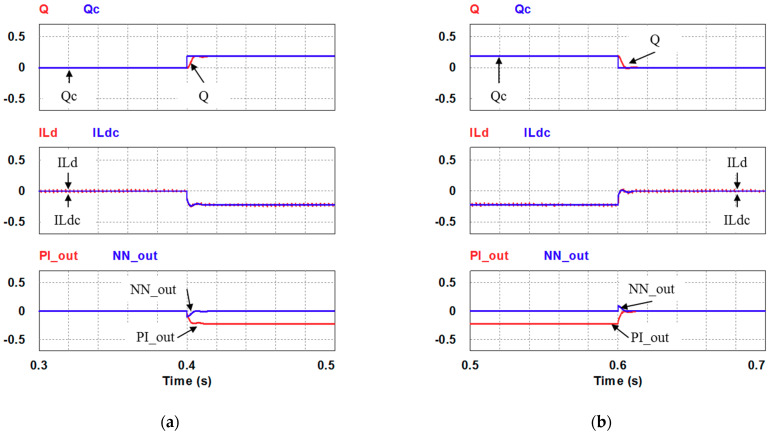
Detailed views of Figure 20a: (**a**) from 0.3 to 0.5 s; (**b**) from 0.5 to 0.7 s.

In this case, the PI controller in the last subsection is joined with the RBFNN controller in the reactive power control loop. Simulation results are presented in Figure 18, Figure 19 and Figure 20b. Figure 18 and Figure 19 show that the phases and amplitudes of three-phase currents are well controlled. In Figure 20a and Figure 21, it can be observed that the rise and fall times for reactive power are 3.2 ms and 3.5 ms, respectively, and the overshoot and undershoot percentages are improved to 5% and 6%, respectively. As can be seen, the main purpose of the RBFNN controller in this case is to suppress the overshoots and undershoots. In Figure 20b, it can be observed that the accumulated reactive power control error is 2.44 m at the end of the simulation.

## 5. Hardware Prototype Implementation 

**Figure 22:**
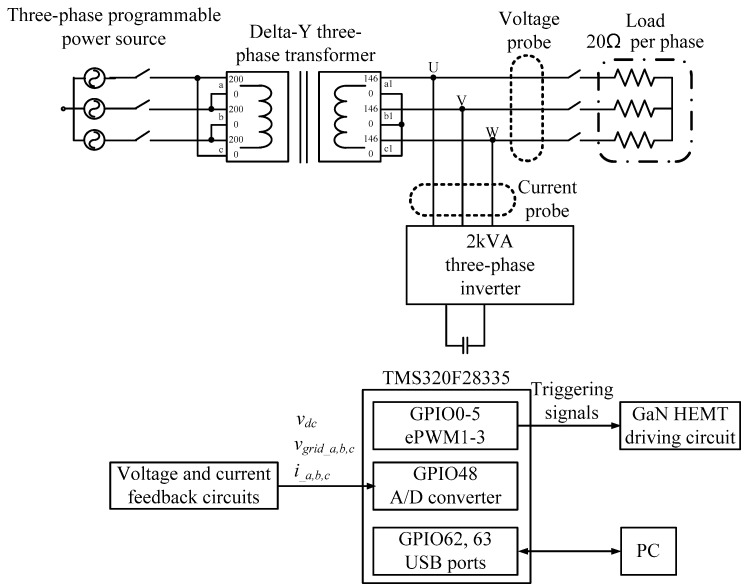
Conceptual diagram of the hardware implementation system.

**Figure 23:**
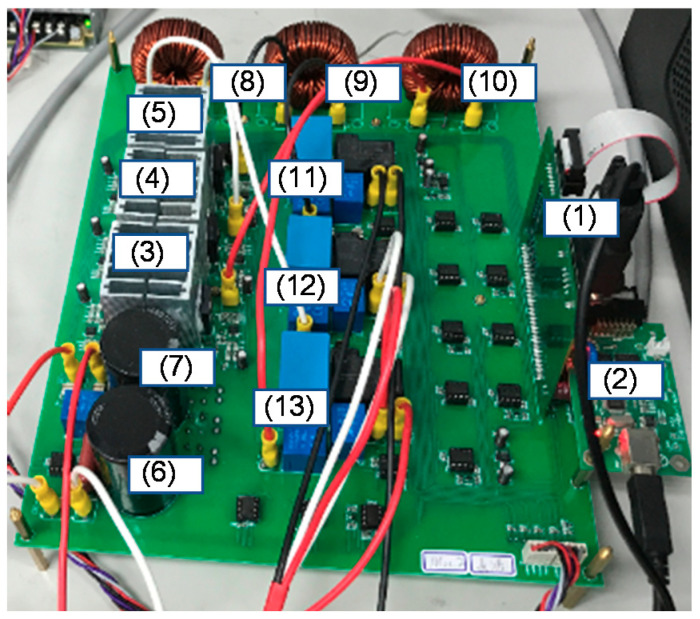
Photo of GaN HEMTs-based three-phase STATCOM hardware prototype.

**Figure 24:**
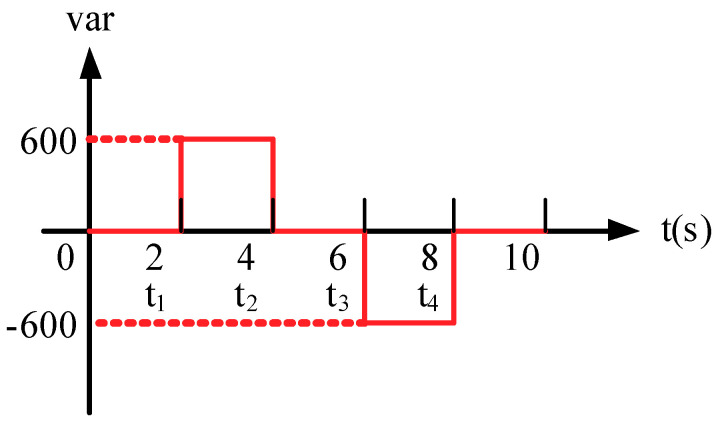
Implementation scenario: sequence diagram of reactive power command.

In order to verify the simulated results of the proposed GaN HEMTs-based STATCOM system and the control schemes, this section presents the hardware implementation of the proposed system using previously simulated scenarios. The schematic block diagram of the hardware implementation is illustrated in Figure 22, where the related specifications are listed in Table 4. The proposed three-phase STATCOM was constructed using GaN HEMT TPH3207 by Transphorm (Goleta, CA, USA). The gate driver adopted for driving the GaN HEMTs was Si8271 by Silicon Labs (Austin, TX, USA). A programmable three-phase power source and a delta-Y 200V/146V transformer emulated the healthy balanced grid. DSP TMS320F28335 was used to provide efficiency and flexibility in controller design. A photograph of the constructed GaN-based STATCOM prototype is shown in Figure 23, while the numbered devices are listed in Table 5. For easier waveform observation, the reactive power command was prolonged to 2 s per interval, as shown in Figure 24, where t_1_ = 2 s, t_2_ = 4 s, t_3_ = 6 s, t_4_ = 8 s. Results of hardware implementation of two reactive power controller settings are presented in the following subsections.

### 5.1. Test Results of Implementation Case 1: PI Controller

**Figure 25:**
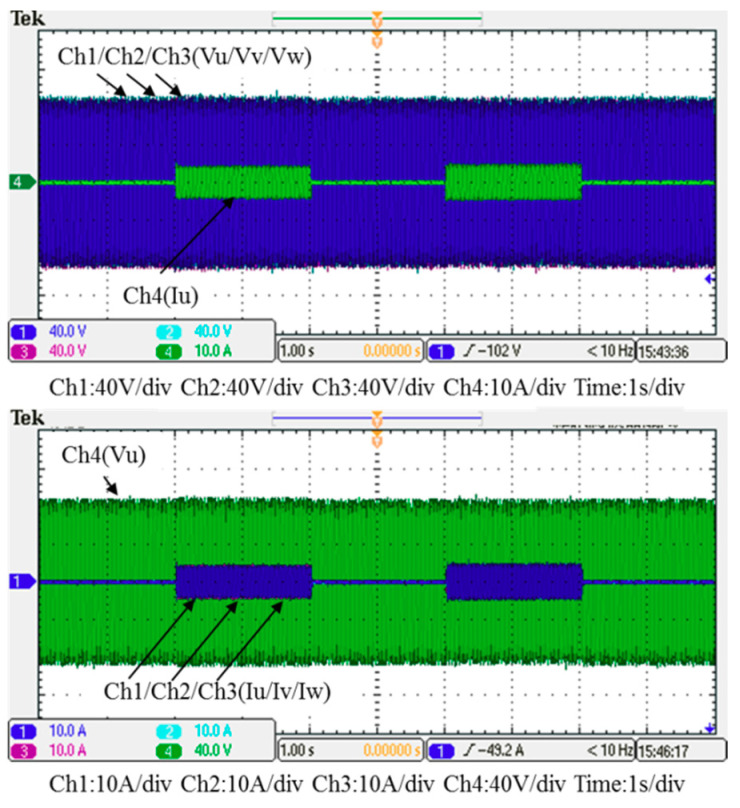
Case 1 implementation result: three-phase voltages and currents.

**Figure 26:**
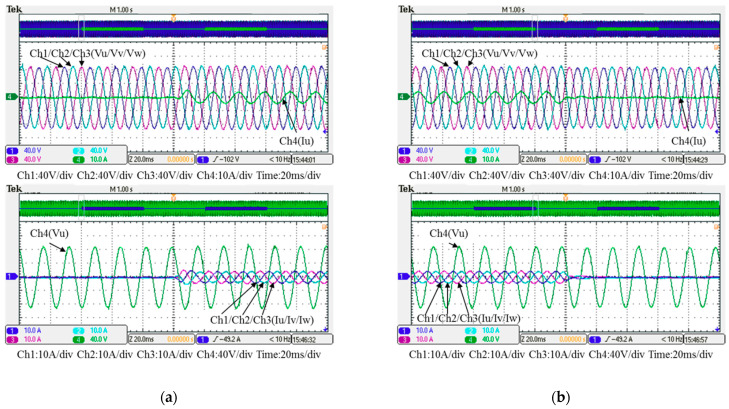
Detailed view of Figure 24: (**a**) near t_1_; (**b**) near t_2_.

**Figure 27:**
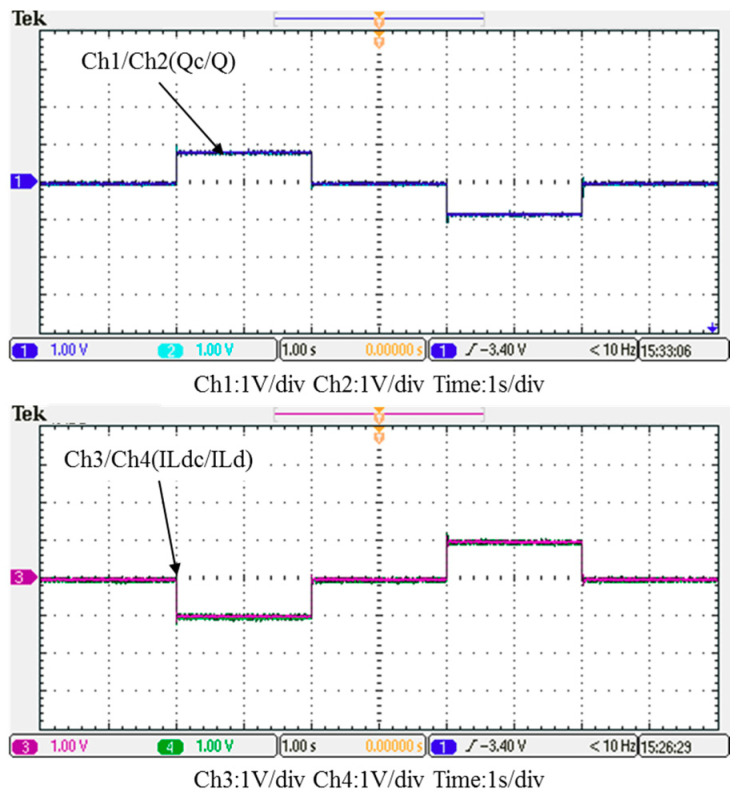
Case 1 implementation result: feedbacks and commands of reactive power and inductor current (all control signals are in V).

**Figure 28:**
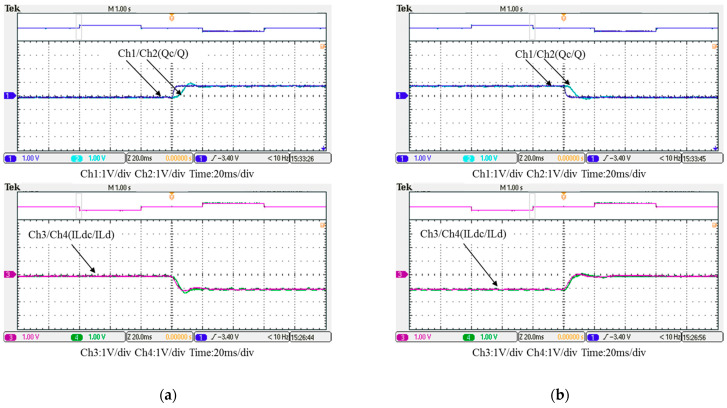
Detailed view of Figure 31: (**a**) near t_1_; (**b**) near t_2_.

**Figure 29:**
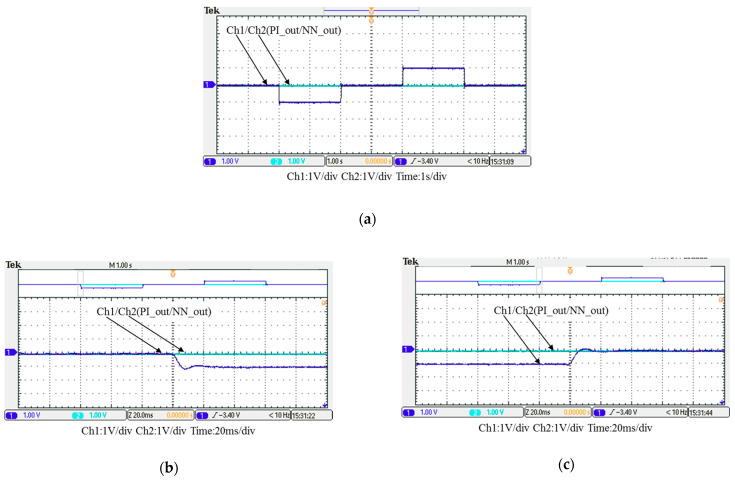
Case 1 implementation result: reactive power controller outputs: (**a**) full view; (**b**) detailed view near t_1_; (**c**) detailed view near t_2_ (all control signals are in V).

In this case, only the PI controller is activated in the reactive power control loop. Measured results are presented in Figure 25, Figure 26, Figure 27, Figure 28 and Figure 29. Figure 25 and Figure 26 show that the phases and amplitudes of three-phase currents are well controlled. In Figure 27 and Figure 28, it can be observed that the rise and fall times for reactive power were 4.3 ms and 4.68 ms, respectively, and the overshoot and undershoot percentages were 24% and 14%, respectively. In Figure 29, it can be observed that the PI controller was able to control the reactive power but the dynamic performance was not sufficient.

### 5.2. Test Results of Implementation Case 2: PI Controller + RBFNN Controller

**Figure 30:**
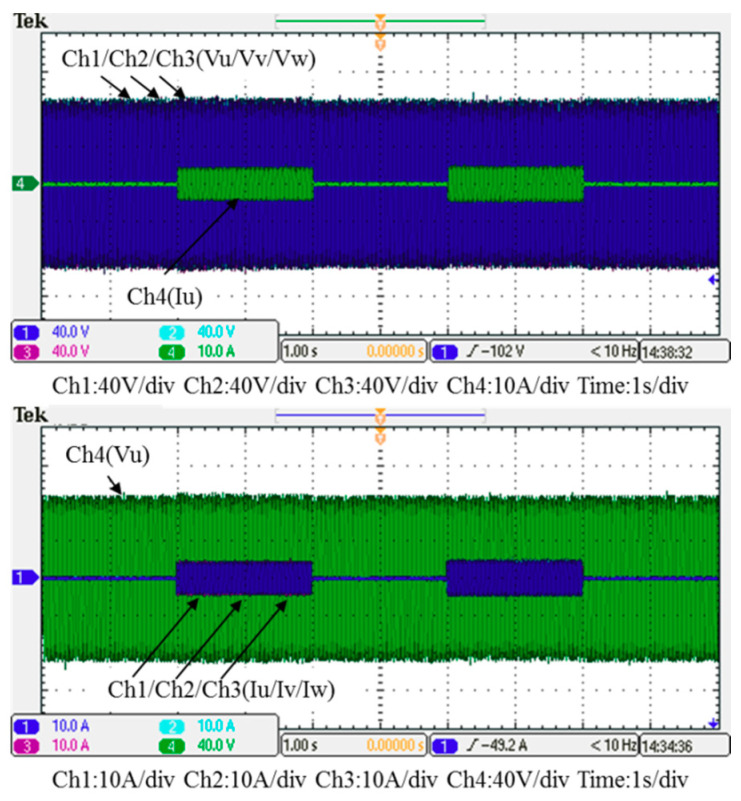
Case 2 implementation result: three-phase voltages and currents.

**Figure 31:**
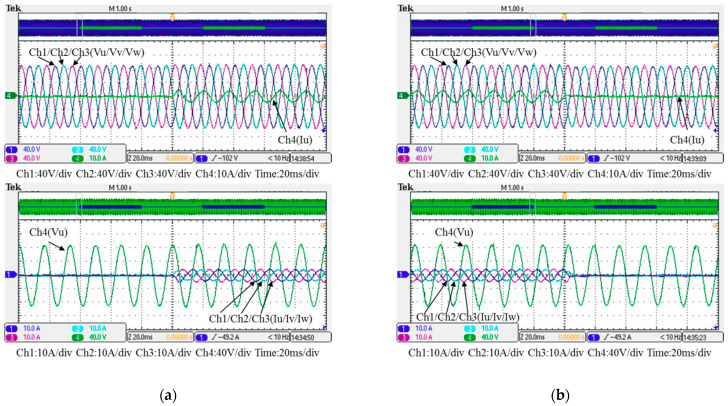
Detailed view of Figure 29: (**a**) near t_1_; (**b**) near t_2_.

**Figure 32:**
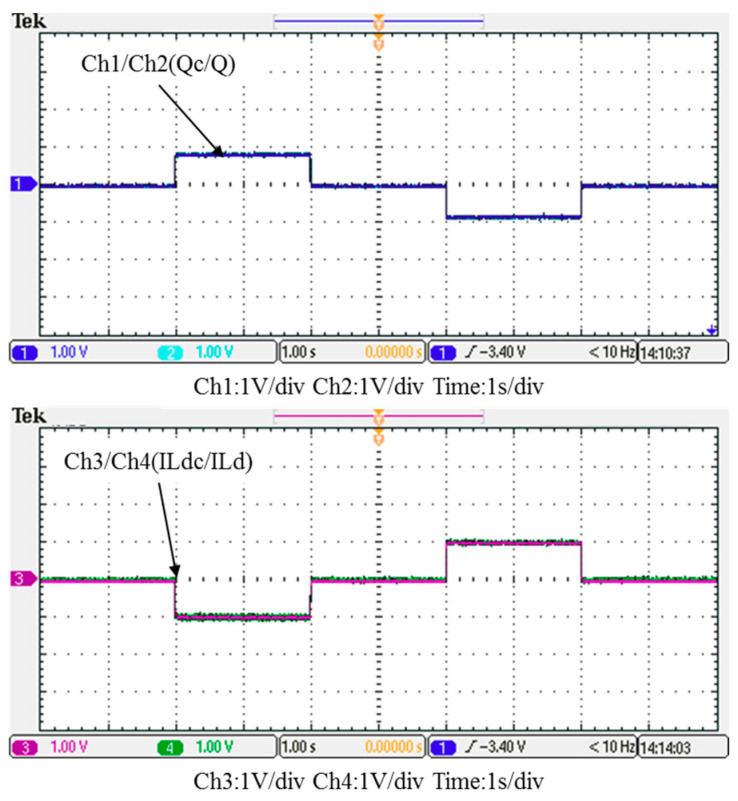
Case 2 implementation result: feedbacks and commands of reactive power and inductor current (all control signals are in V).

**Figure 33:**
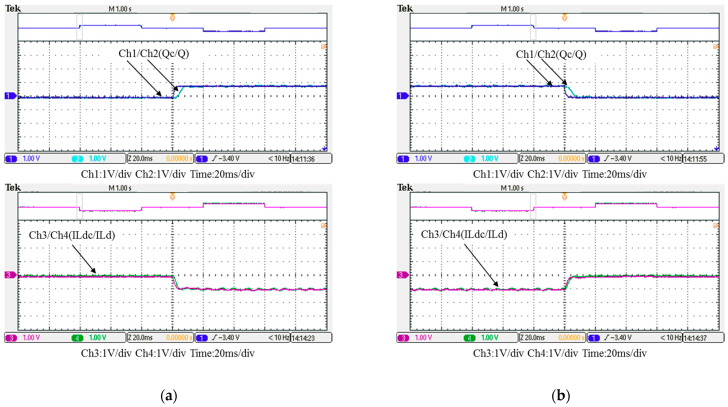
Detailed view of Figure 31: (**a**) near t_1_; (**b**) near t_2_.

**Figure 34:**
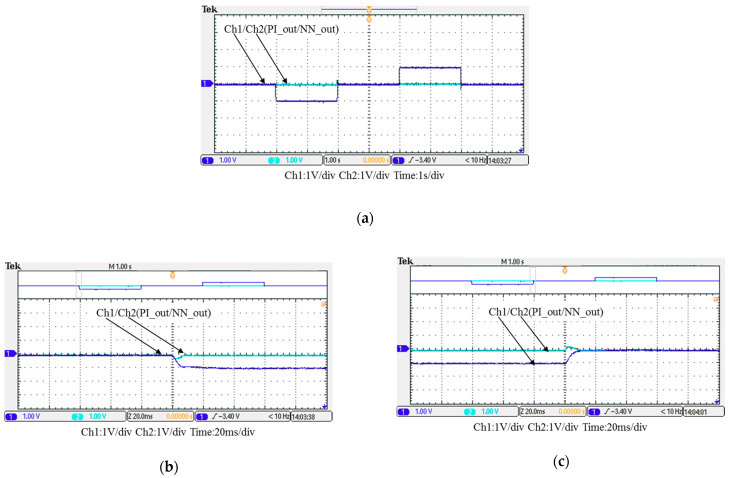
Case 2 implementation result: reactive power controller outputs: (**a)** full view; (**b**) detailed view near t_1_; (**c**) detailed view near t_2_ (all control signals are in V).

In this case, the PI controller in the last subsection is joined with the RBFNN controller in the reactive power control loop. Measured implementation results are presented in Figure 30, Figure 31, Figure 32, Figure 33 and Figure 34. Figure 30 and Figure 31 show that the phases and amplitudes of three-phase currents were well controlled. In Figure 32 and Figure 33, it can be observed that the rise and fall times for reactive power were shortened to 3.52 ms and 4.64 ms, and the overshoot and undershoot percentages were successfully improved to 4% and 2%, respectively. In Figure 34, it can be observed that the RBFNN controller successfully suppressed the overshoots and undershoots.

## 6. Discussion and Conclusions

The results of all simulated and 2kVA hardware implemented scenarios are highlighted in Table 6. It can be observed that combining the PI controller with RBFNN controller forming a hybrid, nonlinear control scheme can effectively improve the dynamic performance of reactive power control, especially in terms of regulation speed and overshoot/undershoot suppression. Therefore, we can conclude that the proposed GaN HEMTs-based three-phase STATCOM taking the advantages of better material features of WBG switching devices and the advanced control scheme on both trained RBFNN and conventional PI controllers is a highly effective design example.

It is important to note that the need to improve the performance of various power converters has driven researchers around the world to investigate on new WBG switching devices and their applications. It has been well proved that the WBG switching devices, including GaN HEMTs and SiC MOSFETs, offer superior performance to that of conventional Si-based switching devices, including higher blocking voltage, current, switching frequency, efficiency, and operating temperature. However, in various design cases reported in open literature, GaN devices tend to dominate low- to mid-voltage and low- to mid-power converter applications, while SiC devices are suitable for higher-voltage and higher-power converter applications. In this aspect, this paper has demonstrated a GaN-based three-phase STATCOM functioning as a fast reactive power regulator. The proposed dual-loop control architecture using SRF consists of inner loop, type II current controllers and outer loop, DC link voltage, and reactive power PI controllers. In addition, an RBFNN controller is designed for constructing a hybrid reactive power control scheme to improve the tracking speed and dynamic performance of the GaN HEMTs-based STATCOM. Both simulation and measured hardware implementation results have verified the feasibility and effectiveness of the proposed design case.

## Figures and Tables

**Table 1:** System specifications.

Item	Value
Grid voltage, $V_{grid\_abc}$	63.5 V_rms_
Grid frequency	60 Hz
Inverter capacity	2 kVA
DC link voltage, $V_{dc}$	200 V
DC link capacitor, $C_{dc}$	1360 μF/400 V
Filter inductor, $L_{f}$	1 mH
Filter capacitor, $C_{f}$	10 μF
DC voltage variation limit	1%
Switching frequency	100 kHz
Carrier amplitude	5 V
DC voltage sensing factor, $k_{vd}$	0.012
AC voltage sensing factor, $k_{v}$	0.0062
AC current sensing factor, $k_{s}$	0.05

**Table 2:** Reactive power controller setting.

Case	RBFNN Controller
1	Inactivated
2	Activated

**Table 3:** Equivalent values of control signals.

Item	Sensing Factor	Equivalent Value
Reactive power & Reactive power controller output	*k_s_* ∗ *k_v_* (0.00031)	1 V→3225.8 VAR
Inductor current	*k_s_* (0.05)	1 V→20 A

**Table 4:** Specifications and parameters of hardware implementation.

Item	Value/Part Number
DSP	TMS320F28335
Load	20 Ω per phase
Delta-Y three-phase transformer	200 V/146 V
GaN HEMT	TPH3207
Gate driver	Si8271
Grid voltage, *v_grid_abc_*	63.5 V_rms_
Grid frequency	60 Hz
Inverter capacity	2 kVA
DC link voltage, *V_dc_*	200 V
DC voltage sensing factor, *k_vd_*	0.012
AC voltage sensing factor, *k_v_*	0.0062
AC current sensing factor, *k_s_*	0.05

**Table 5:** Devices in Figure 23.

Number	Device	Value/Part Number
(1)	DSP	TMS320F28335
(2)	DSP interface	N/A
(3)–(5)	GaN HEMT pairs	TPH3207
(6) & (7)	DC link capacitors	680 μF/400 V
(8)–(10)	Filter inductors	1 mH
(11)–(13)	Filter capacitors	10 μF/300 V

**Table 6:** Result comparison.

Case	Controllers	Rise time (ms)		Fall time (ms)		Overshoot (%)		Undershoot (%)	
		Sim.	Imp.	Sim.	Imp.	Sim.	Imp.	Sim.	Imp.
1	PI	3.469	4.3	3.558	4.68	20	24	20	14
2	PI + NN	3.2	3.52	3.5	4.64	5	4	6	2

## Data Availability

No new data were created or analyzed in this study. Data sharing is not applicable to this article.
